# Latent Class Trajectory and Growth Mixture Models in the Study of Physical Resilience

**DOI:** 10.1093/geroni/igaa057.3030

**Published:** 2020-12-16

**Authors:** Carl Pieper, Jane Pendergast, Megan Neely

**Affiliations:** 1 Center on Aging and Human Development, DUMC, Durham, North Carolina, United States; 2 Duke University, Durham, North Carolina, United States

## Abstract

After a stressor, individuals may experience different trajectories of function and recovery. One potential explanation for this variation is differing trajectories may be indicators of differing classes or levels of resilience to the stressor. Latent Class Trajectory (LCTA) and Growth Mixture models (GMM) are two similar approaches used to discover the number and types of trajectories in a study population. Class membership may determine the shape and level of recovery, which may be predicted by individual characteristics. In this talk, we present some insights to using these models to successfully identify the number of classes of trajectories, membership of trajectory classes, and the functional form of the trajectory. We will identify methods for deciding class enumeration, indices for assessing fit quality, and, importantly, the importance of proper model specification. Real life and simulated examples will be shown to compare and contrast differences between GMM and LCTA results. Part of a symposium sponsored by Epidemiology of Aging Interest Group.

